# Structure and sucrose binding mechanism of the plant SUC1 sucrose transporter

**DOI:** 10.1038/s41477-023-01421-0

**Published:** 2023-05-15

**Authors:** Laust Bavnhøj, Jan Heiner Driller, Lorena Zuzic, Amanda Dyrholm Stange, Birgit Schiøtt, Bjørn Panyella Pedersen

**Affiliations:** 1 Department of Molecular Biology and Genetics, Aarhus University, Aarhus, Denmark; 2 Department of Chemistry, Aarhus University, Aarhus, Denmark

**Keywords:** Plant molecular biology, Plant sciences

## Abstract

Sucrose import from photosynthetic tissues into the phloem is mediated by transporters from the low-affinity sucrose transporter family (SUC/SUT family). Furthermore, sucrose redistribution to other tissues is driven by phloem sap movement, the product of high turgor pressure created by this import activity. Additionally, sink organs such as fruits, cereals and seeds that accumulate high concentrations of sugar also depend on this active transport of sucrose. Here we present the structure of the sucrose–proton symporter, *Arabidopsis thaliana* SUC1, in an outward open conformation at 2.7 Å resolution, together with molecular dynamics simulations and biochemical characterization. We identify the key acidic residue required for proton-driven sucrose uptake and describe how protonation and sucrose binding are strongly coupled. Sucrose binding is a two-step process, with initial recognition mediated by the glucosyl moiety binding directly to the key acidic residue in a stringent pH-dependent manner. Our results explain how low-affinity sucrose transport is achieved in plants, and pinpoint a range of SUC binders that help define selectivity. Our data demonstrate a new mode for proton-driven symport with links to cation-driven symport and provide a broad model for general low-affinity transport in highly enriched substrate environments.

## Abstract (web summary)

The crystal structure of the *Arabidopsis* sucrose–proton symporter SUC1 is presented at 2.7 Å resolution. This allows the identification of a proton-driven symport process for SUC1 that may help in understanding how low-affinity transport can occur in enriched substrate environments.

## Main

Sucrose is essential for plant growth and development, serving as the central plant metabolite, with additional associated roles as a signalling molecule^1–3^. In most plant species, sucrose is the main form of assimilated carbon produced during photosynthesis. Long-distance sucrose distribution from the green source tissues, generally leaves, to energy-demanding sink tissues is mediated by phloem^4^. Here sugar transporters from two families, the sucrose transporter family (named the SUT/SUC family) and the SWEET family, play central roles^5–8^. Whereas the SWEET facilitators efflux sugars, active loading depends on SUCs that use the plasma membrane proton motive force to drive uptake of sucrose for accumulation, with concentrations reaching as high as 1.3 molar in the phloem^9–14^. Phloem sap movement is the product of the high turgor pressure created by this SUC activity^6,15^. In addition, sink organs, such as fruits and seeds, that accumulate high concentrations of sugar in developmental stages also depend on the active transport of sucrose by SUCs^15,16^.

SUCs are part of the Glycoside-Pentoside-Hexuronide (GPH):cation symporter family that belongs to the Major Facilitator Superfamily (MFS)^17,18^. Members of the GPH family are known to transport a wide range of substrates, from small soluble molecules such as glucose, melibiose or sucrose to large amphiphilic lysolipids, normally coupled to transport of monovalent cations^19–21^. SUCs hold a unique position in the diverse GPH family by being proton-coupled transporters, and have very low sequence identity to other known branches (<16%).

**Extended Data Fig. 1:**
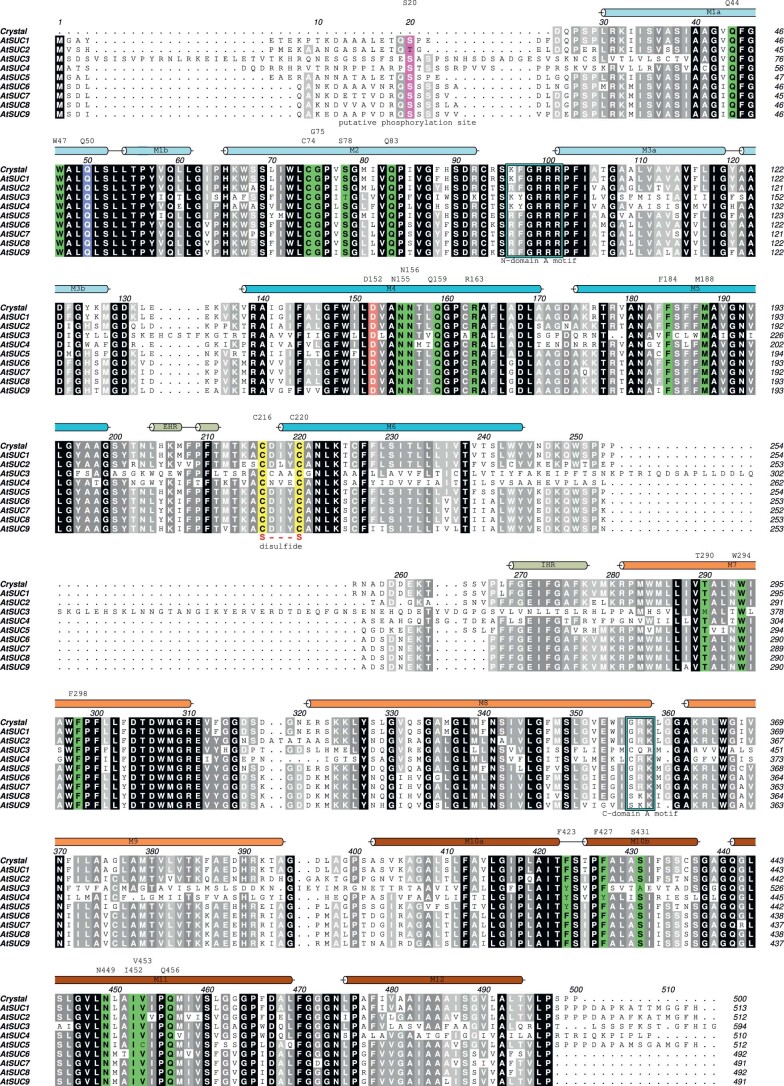
Multiple sequence alignment of the *Arabidopsis thaliana* Sucrose Transporters. Alignment between *A. thaliana* SUC1 (accession number Q39232), SUC2 (accession number Q39231), SUC3 (accession number O80605), SUC4 (accession number Q9FE59), SUC5 (accession number Q9C8X2), SUC6 (accession number Q6A329), SUC7 (accession number Q67YF8), SUC8 (accession number Q9ZVK6), SUC9 (accession number Q9FG00). Conserved residues are highlighted with gray-scale, where black is perfectly conserved. Coloured tubes represent α-helices found in the N domain (cyan), EHR and IHR (grey) and C domain (orange). Key residues are numbered above the α-helix markings. Key residues are highlighted in green, except proton donor/acceptor pair in red (Asp152) and blue (Gln50). The cysteines forming the disulfide bridge between EHR and M6 are highlighted in yellow. The A motifs that denote the MFS superfamily are highlighted in light blue. Post-translational phosphorylation site at the N-terminal^92^ is coloured light magenta. Mutating this site residue (Ser20) to alanine in SUC1 did not affect uptake in oocytes compared to wild type (Extended Data Fig. 7b).

Despite their key role in fundamental processes of plant physiology, the working mechanism of SUCs remains unknown. It is unclear how SUCs recognize sucrose and how transport is proton coupled. Possessing the ability to release sucrose into environments with extremely high sucrose concentrations, the sucrose binding site must have unique features; indeed, its coupling to the driving proton gradient must be tight and well coordinated. *Arabidopsis thaliana* has nine SUC transporters (SUC1–9), with SUC1 being among the first sucrose–H^+^ symporters identified (Extended Data Fig. 1)^22–28^. SUC1 is predominantly expressed in reproductive organs, trichomes and roots, where it localizes in the plasma membrane and is required for proper pollen function^24,29–33^.

Here we present the structure of *A. thaliana* SUC1 along with comprehensive biochemical characterization and molecular dynamics (MD) simulations. Our work pinpoints elements key to understanding proton-coupled sucrose transport by explaining substrate recognition and how protons are directly coupled to sucrose transport in a unique mechanism. This mechanism allows for sucrose binding and release in environments with high molar sucrose concentrations, which is vital for proper phloem function, transport into apoplastically isolated tissues and plant growth.

## Results

### Activity and overall structure of SUC1

**Fig. 1:**
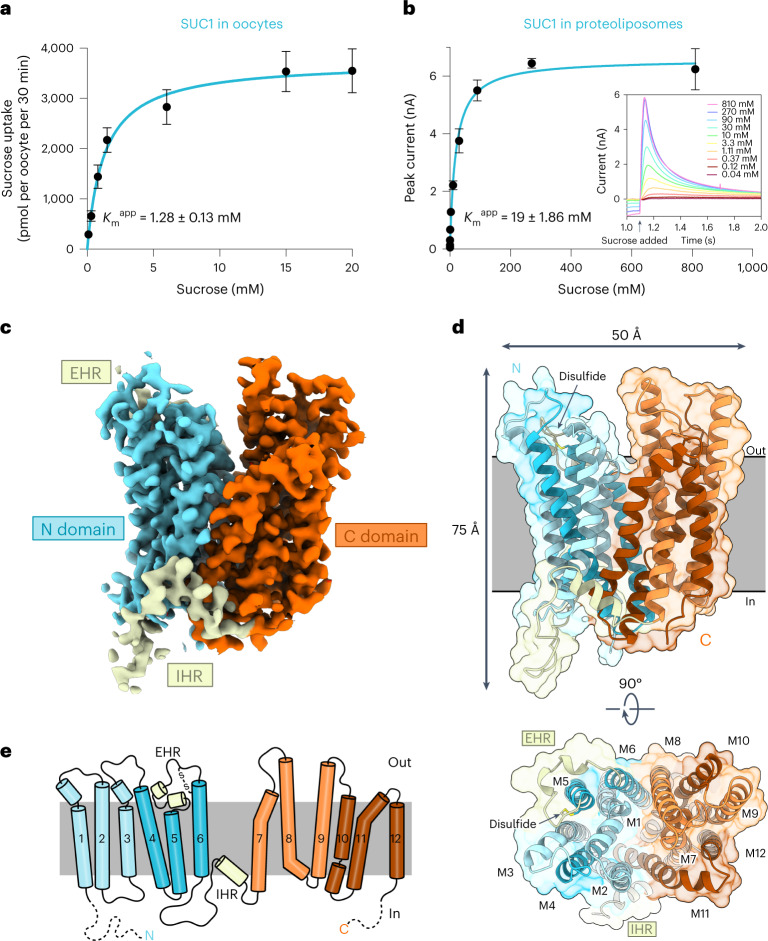
Activity and structure of SUC1 in the outward open conformation. **a**, Oocyte uptake assay for SUC1 at pH 5.5 with Michaelis–Menten nonlinear fit. Data points are mean ± s.d.; points represent biologically independent measurements (*n* = 4 for concentrations of 0.8, 1.5, 6 and 15 mM; *n* = 6 for a concentration of 20 mM; *n* = 9 for concentrations of 0.1 and 0.3 mM). **b**, Peak currents determined by SSM-based electrophysiology on SUC1 proteoliposomes at symmetrical pH 5.5. Transport can be described by Michaelis–Menten kinetics. Data points are mean ± s.d. (*n* = 3 biological independent experiments). Insert shows raw current traces at a range of sucrose concentrations. **c**, The 2.7 Å electron density map of SUC1 (2mFo-DFc map contoured at 1*σ*). Density corresponding to the N and C domain are coloured cyan and orange, respectively. EHR and IHR domains are coloured pale yellow. **d**, Side view of the structure of SUC1 in the plane of the plasma membrane, coloured according to domains. Bottom, a top view from the extracellular side with M1–M12 labelled. The EHR–M6 connecting disulfide bridge is shown. **e**, Topology of SUC1. Disordered N-terminal (residues 1–24) and C-terminal (residues 501–513) ends are shown as dashes. Source data

**Extended Data Fig. 2:**
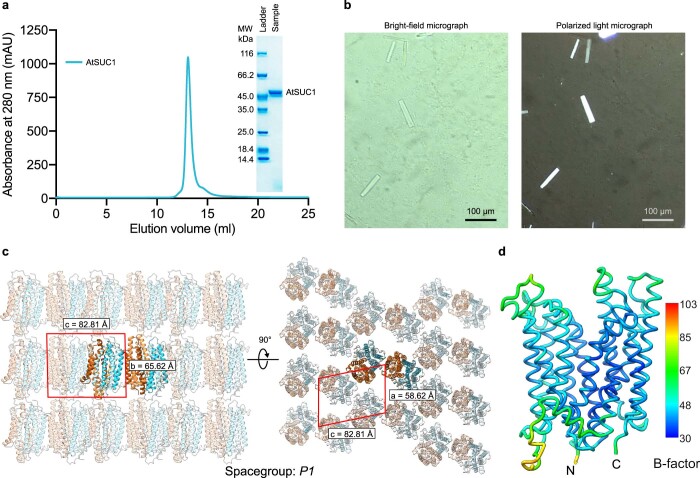
Expression and crystal packing of SUC1. **a**, Size-exclusion chromatography profile of SUC1 and representative SDS-PAGE gel of purified SUC1 protein. Independent experiments have been repeated three times with similar results. **b**, Polarized light photo and bright field photo of SUC1 crystals in lipidic cubic phase setup. Independent experiments have been repeated three times with similar results. **c**, Asymmetric unit and crystal packing of SUC1. **d**, The backbone of SUC1 colored by the atomic displacement factor (B-factor) with a rainbow gradient from low/blue (29.91 Å^2^) to high/red (103.48 Å^2^) with a mean of 47.10 Å^2^.

**Extended Data Fig. 3:**
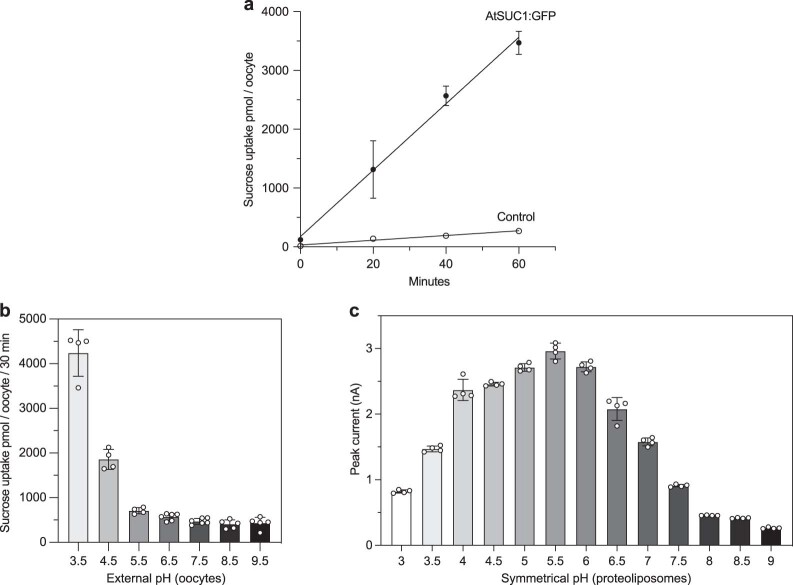
Uptake of sucrose by SUC1 and pH dependence. **a**, Uptake of sucrose into SUC1-expressing oocytes (black circles) and water-injected oocytes (empty circles) at an initial outside concentration of 1 mM sucrose at pH 5.5. Data points are mean ± s.d.; *n* = 4 oocytes and each oocytes represent individual measurements. **b**, Oocyte uptake assay display classical pH dependence that scales linearly with external proton concentration over the viable range of the experimental setup. Transport was measured at pH 3.5–9.5 in phosphate buffer. All measurements were performed in presence of 1 mM sucrose. Bars are mean ± s.d.; Data points are individual experiments (*n* = 4 (pH 3.5,4.5,5.5), *n* = 5 (pH 8.5,9.5), *n* = 6 (pH 6.5,7.5). **c**, Peak currents using SSM-electrophysiology for the SUC1 WT proteoliposomes elicited by addition of 10 mM sucrose at indicated symmetrical pH (pH_in_ = pH_out_). Bars are mean ± sd and *n* = 4. The points are individual measurements. Source data

To examine the transport mechanism of SUCs, we screened a range of SUC transporters and identified that *A. thaliana* SUC1 is stable and well expressed in *Saccharomyces cerevisiae* (Extended Data Fig. 2a–d). In oocyte uptake assays, SUC1 shows robust uptake of sucrose with an apparent Michaelis constant (*K*_m_^app^) of 1.28 mM (Fig. 1a and Extended Data Fig. 3a), comparable with previous results^6,34^. Purified SUC1 protein was reconstituted into liposomes and transport was measured using capacitive coupling by solid supported membrane (SSM) electrophysiology. This showed a similar robust but low affinity for sucrose in vitro, with a *K*_m_^app^ (SSM-based electrophysiology assays in Methods) of 19 mM at pH 5.5 (Fig. 1b). We found a strong pH dependence, with transport being stimulated by increased proton concentration in vivo and with a functional optimum in vitro at symmetrical pH 5.5 (Extended Data Fig. 3b,c).

**Extended Data Fig. 4:**
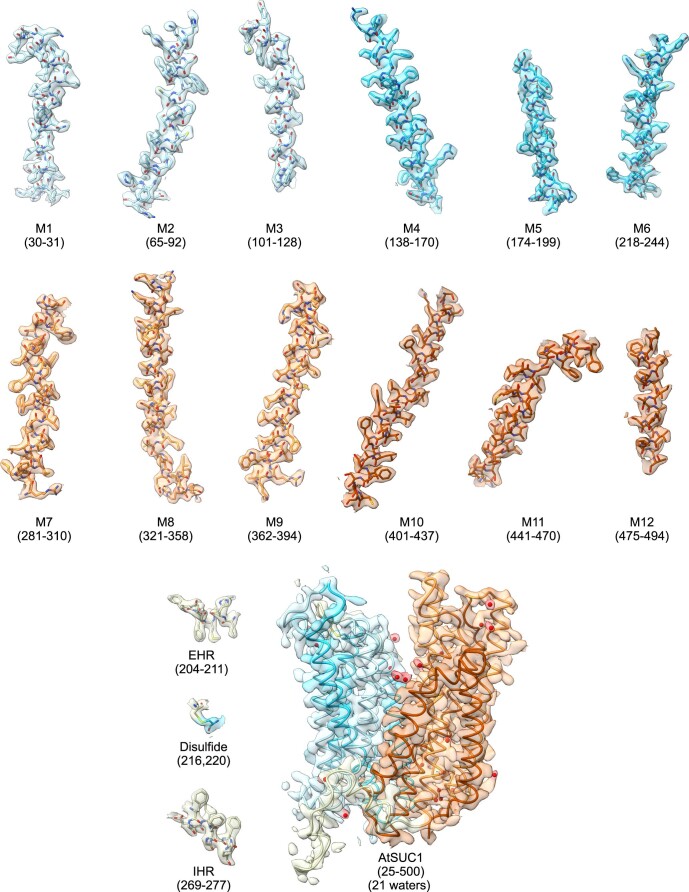
Fit of electron density for SUC1 outward open structure. Weighted 2mFo-DFc density at 1σ of SUC1 outward open structure (monomer, chain A) with the final model elements overlaid, including M1 to M12, EHR helix, C216-C220 disulfide bridge, IHR helix and full model shown as licorice with waters (red dots) included.

**Extended Data Fig. 5:**
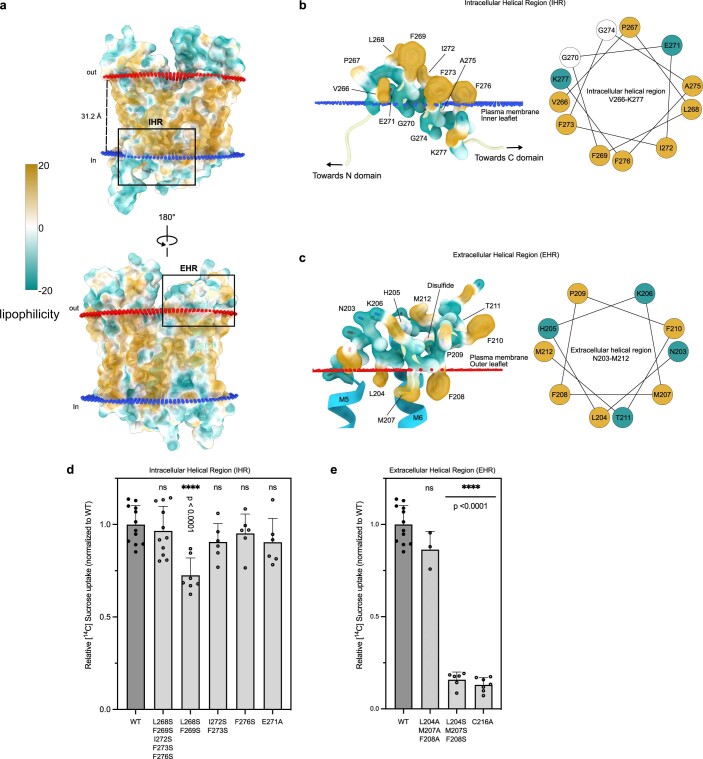
Amphipathic nature of helical regions at the plasma membrane leaflets. **a**, Side views of the SUC1 structure with molecular lipophilicity potential (MLP) shown as molecular surface and coloured from dark cyan (most hydrophilic) to dark goldenrod (most lipophilic). Insets highlight helical regions at the plasma membrane leaflets. Membrane boundaries (outer leaflet; blue, inner leaflet; red) were calculated using the PPM web server^93^. **b**, Amphipathic intracellular helical region (IHR) shown as cartoon with labelled residues as sticks (left) coloured according to lipophilicity as in panel A and Edmundson wheel projection diagram of the residues of helical region with hydrophobic residues facing the inner leaflet of the plasma membrane and polar residues facing the cytoplasm (right). **c**, Amphipathic extracellular helical region (EHR) shown as cartoon and labeled residues as sticks (left) colored according to lipophilicity as in panel A and Edmundson wheel projection diagram of the residues of helical region with hydrophobic residues facing the outer leaflet of the plasma membrane and polar residues facing the apoplast. **d**, Oocyte uptake assay for SUC1 mutants targeting the IHR. Uptake activities are normalized to that of the wild type. Control is water injected oocytes. Bars are mean ± s.d. Data points are individual experiments (*n* = 6 (I272S/F273S,E271A,F276S), *n* = 7 (L268S/F269S), *n* = 11 (L268S/F269S/I272S/F273S/F276S), *n* = 16(WT)). P values for differences between wild type and variants was from a one-way ANOVA analysis followed by Dunnett’s multiple comparisons test (*p* = 0.9145 (L268S/F269S/I272S/F273S/F278S), *p* = 0.2915 (I272S/F273S), *p* = 0.8544 (F276S), *p* = 0.6264 (E271A)). **e**, Oocyte uptake assay for SUC1 mutants targeting the EHR. Uptake activities are normalized to that of the wild type. Control is water injected oocytes. Bars are mean ± s.d. Data points are individual experiments (*n* = 3 (L204S/M207S, F208S), *n* = 6 (L204A/M207A/F208A), *n* = 7 (C216A), *n* = 16 (WT)). P values for differences between wild type and variants was from a one-way ANOVA analysis followed by Dunnett’s multiple comparisons test (*p* = 0.4290 (L204A/ M207A/F208A)). Source data

**Extended Data Fig. 6:**
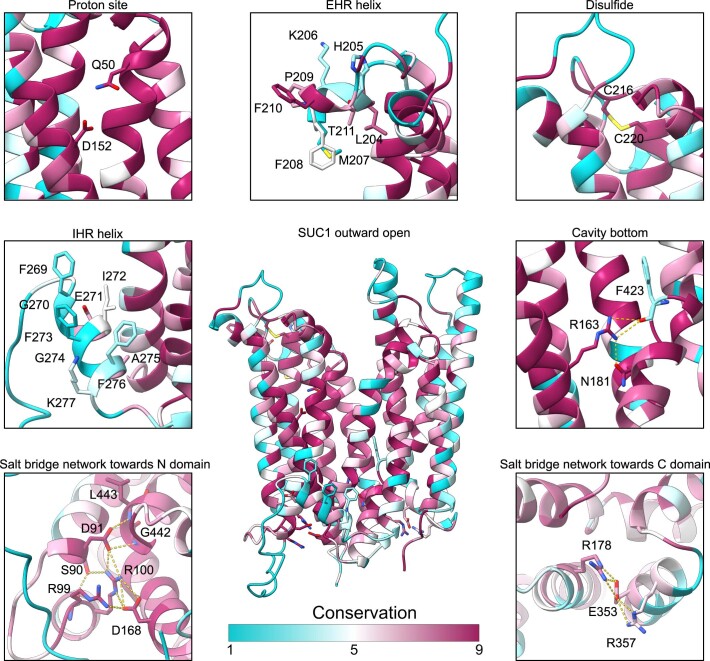
Conservation analysis of SUC1 structure. Conservation of the residues in SUC1 (shown as cartoon) between SUC sequences (with 35–95% seq. ID to SUC1) across plant species as obtained by ConSurf. The residues (shown as sticks) are coloured from variable (cyan) to fully conserved (magenta).

We proceeded to solve the structure of SUC1 using crystallography at 2.7 Å resolution (Fig. 1c and Extended Data Fig. 2a–d). In the asymmetrical unit, SUC1 forms a non-physiological inverted dimer, with both monomers in the same conformation (root mean square deviation (r.m.s.d.) of Cɑ atoms = 0.1 Å). Excellent map density allowed the construction of a final atomic model containing residues 25–500 (of 513 residues) with a free *R* factor (*R*_free_) of 29.3% (Fig. 1c, Extended Data Fig. 4 and Supplementary Table 1). Overall, SUC1 adopts the canonical MFS transporter fold with twelve transmembrane helices arranged into two pseudosymmetrical halves consisting of two six-helix domains, the N-terminal domain (N domain, M1–M6) and C-terminal domain (C domain, M7–M12) (Fig. 1d,e). The two domains are connected by a short cytoplasmic linker region of 26 residues, which are well defined in the experimental map. Of note, the cytoplasmic linker contains a 9 residue amphipathic ɑ-helix (named intracellular helical region (IHR)) (Fig. 1c–e and Extended Data Figs. 4 and 5a,b). Extracellularly, a short helical region (named extracellular helical region (EHR)), which also has clear amphipathic properties, is present in the loop connection between M5 and M6. The EHR is anchored to the side of the N domain by a disulfide bridge between the strictly conserved residues Cys216 and Cys220 (Fig. 1d and Extended Data Figs. 4, 5a,c and 6). We explored the roles of the IHR and EHR by mutagenesis and found that transport activity in oocytes appears indifferent to mutagenesis of the hydrophobic residues in the IHR (Extended Data Fig. 5b,d). However, the EHR at the extracellular side is very sensitive to changes. Disrupting the short EHR–M6 link by mutating Cys216 of the disulfide bridge results in loss of transport. Mutation of the three residues (Leu204, Met207 and Phe208) of the EHR, which appear buried in the membrane leaflet, to polar serine residues gives a similar loss of transport, implying an important role during transport for this extracellular region (Extended Data Fig. 5e).

**Fig. 2:**
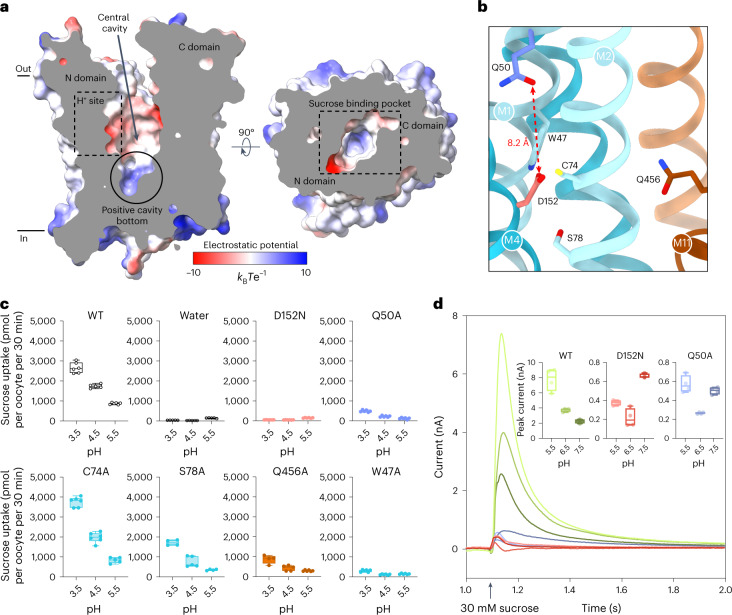
The identified proton-binding site in SUC1. **a**, Slab view of SUC1 showing the outward open conformation viewed from the membrane plane (left) or from the extracellular side (right). Dashed boxes refer to regions of the H^+^-binding site and sucrose binding pocket. Surface is coloured by electrostatic potential (*k*_B_*T* e^−1^). **b**, Close-up view of the proton-binding site shown in **a**. The distance between the residue pair constituting the proton site (Asp152 (red) and Gln50 (blue)) is indicated by the dashed arrow, with 8.2 Å being the shortest distance (from Asp152 ε-oxygen to Gln50 ε-oxygen/nitrogen). **c**, Oocyte uptake assay for SUC1 mutants targeting the proton-site pocket at three different external pH values. The box extends from the 25th to 75th percentile, and the median is shown. The whiskers extend to minimum and maximum value. Points represent biologically independent experiments (*n* = 4 for S78A (pH 3.5 and 5.5) and Q456A (pH 3.5); *n* = 5 for WT (pH 4.5), water (pH 3.5, 4.5 and 5.5), C74A (pH 5.5), S78A (pH 4.5), and Q456A (pH 4.5); and *n* = 6 for WT (pH 3.5 and 5.5), W47A (pH 3.5, 4.5 and 5.5), Q50A (pH 3.5, 4.5 and 5.5), C74A (pH 3.5 and 4.5), D152N (pH 3.5, 4.5 and 5.5) and Q456A (pH 5.5)). Colours correspond to residues in panel **b**. **d**, Raw current traces from SSM-based electrophysiology for WT SUC1 (pale green to dark green), SUC1-D152N (pale red to dark red) or SUC1-Q50A (pale blue to dark blue) proteoliposomes elicited by addition of 30 mM sucrose at the indicated symmetrical pH. Plots depict peak currents. The box extends from the 25th to 75th percentile and the median is shown. The whiskers extend to minimum and maximum value (*n* = 3, data points are individual experiments). WT, wild type. Source data

**Extended Data Fig. 7:**
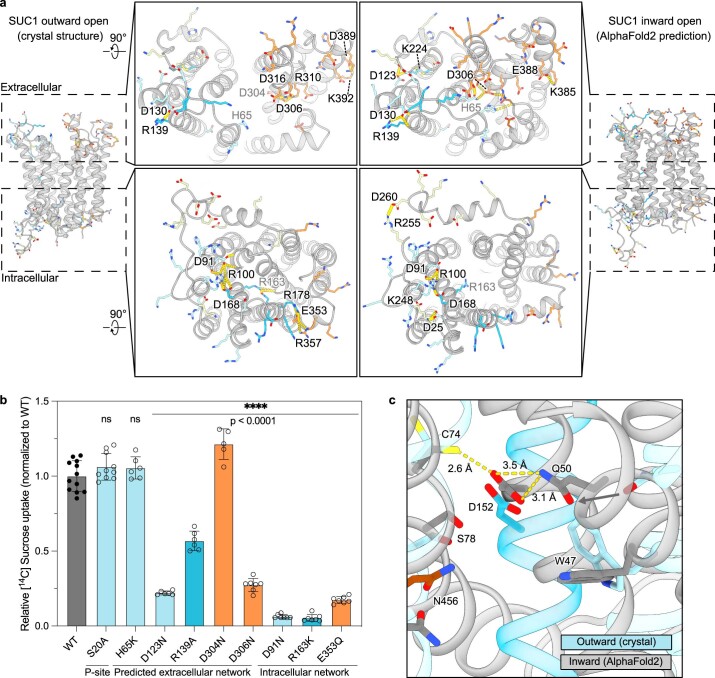
Comparison of SUC1 structure characteristics to computationally predicted inward open model. **a**, Extracellular and intracellular charged residues with labeled residues involved in salt bridge formation (black font) or hydrogen bonds (gray font) in the SUC1 outward open crystal structure and in the AlphaFold2 predicted SUC1 inward open state. Yellow dashes indicate hydrogen bonds (Distance and angle cutoffs for H-bonding as defined by default settings in chimeraX v1.4). **b**, Oocyte uptake assay for SUC1 mutants targeting the hydrogen bonds and salt bridge networks observed in the structure (intracellular network) or predicted (predicted extracellular network), as well as the SUC1 phosphorylation site on Ser20 (P-site). Uptake activities are normalized to that of the wild type. Bars are mean ± s.d. Data points are individual experiments (*n* = 5 (D304N), *n* = 6 (D123N, R139A), *n* = 7 (H65K,D91N,R163K,D306N,E353Q), *n* = 10 (S20A), *n* = 13 (WT)). P values for differences between wild type and variants was from a one-way ANOVA analysis followed by Dunnett’s multiple comparisons test (*p* = 0.1624 (S20A), *p* = 0.9729 (H65K)). Colors correspond to residue position in Fig. 1e. **c**, AlphaFold2 prediction of SUC1 inward open model (grey) superimposed to the experimentally determined SUC1 outward open crystal structure (cyan). Yellow dashes indicate hydrogen bonds and grey arrow indicates major movement of the M1 helix with Gln50 (Distance and angle cutoffs for H-bonding as defined by default settings in chimeraX v1.4). Source data

The structure captures SUC1 in an outward open conformation with a conserved electrostatic network between the N and C domains at the cytosolic side (Extended Data Figs. 6 and 7). A large and clearly defined V-shaped cavity, which spans approximately two thirds of the membrane plane between the N and C domain, is open to the extracellular side (Fig. 2a). Overall, the cavity is electronegative, but it becomes electropositive towards the enclosing bottom. Despite the presence of sucrose during crystallization, no defined density for sucrose binding can be observed in the cavity.

### The proton-binding site of SUC1

**Extended Data Fig. 8:**
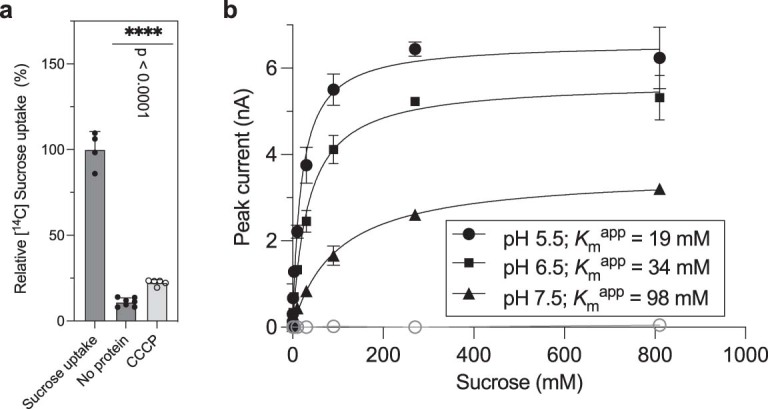
Proton-coupled sucrose transport of SUC1. **a**, Oocyte uptake assay without or with addition of 20x fold (20 mM) CCCP (light grey) at pH 5.5. Water-injected oocytes (no protein) served as control for uptake. P values for differences between control (sucrose uptake) and no protein and CCP was from a one-way ANOVA analysis followed by Dunnett’s multiple comparisons test. P values are shown in the figure. Bars are mean ± sd; Data points are individual experiments (*n* = 4 (sucrose uptake), *n* = 5 (CCCP), *n* = 7 (no protein)). **b**, Peak currents determined by SSM-electrophysiology on SUC1 proteoliposomes at symmetrical pH values as indicated. Transport was described by Michaelis-Menten kinetics by performing nonlinear fit analysis. Grey circles show peak currents from a sensor with prepared with protein empty liposomes. Data points are mean ± s.d. (n = 3 biologically independent experiments). Source data

**Extended Data Fig. 9:**
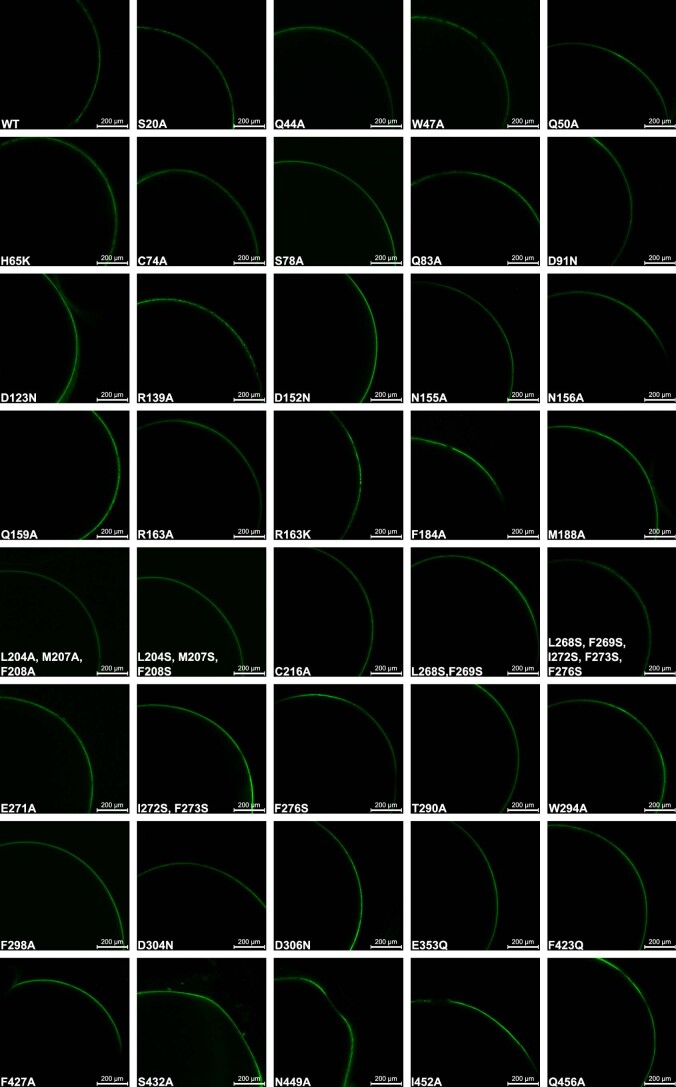
SUC1 mutant localization in oocytes. SUC1 and corresponding mutants harboring a C-terminal GFP show similar localization to the plasma membrane of *Xenopus laevis* oocytes. Independent experiments have been repeated two times with similar results.

The active transport of sucrose by SUC1 is proton driven and sensitive to the proton uncoupler carbonyl cyanide *m*-chlorophenyl hydrazone in oocyte assays (Extended Data Fig. 8a). In transporters, acidic residues buried in the transmembrane region are generally essential for proton transport^35^. In the transmembrane domains of SUC1, the only acidic residue is Asp152(M4), which is strictly conserved in SUCs and therefore the prime candidate as the proton donor/acceptor residue during symport (Fig. 2b and Extended Data Figs. 1 and 6). Asp152 is exposed to the cavity solvent without any close contacts to other residues (Fig. 2b). Mutating Asp152 to an asparagine resulted in loss of transport activity without affecting plasma membrane localization in vivo (Fig. 2c and Extended Data Fig. 9). As expected, this mutant also disrupts the current response otherwise observed for wild-type SUC1 in liposomes, supporting its key role as the central proton donor/acceptor (Fig. 2d and Extended Data Fig. 8b). Another general requirement for transmembrane proton translocation is p*K*_a_ control of the proton donor/acceptor, which is often mediated by a positively charged residue^35–38^. However, there are no obvious candidates for this function in the outward open state of SUC1.

To obtain an inward-facing state, we tried to use different artificial intelligence (AI)-based protein structure prediction tools, but obtained only outward-facing conformations similar to our experimental structure (r.m.s.d. of Cɑ 1.6–2.5 Å). However, in the structure, we identified a network of salt bridges between the N and C domain at the intracellular side that stabilizes the outward open state (Extended Data Figs. 6 and 7a). Disrupting these interactions led to inactive or greatly diminished transport activity (Extended Data Fig. 7b). We inferred that the protein had been stabilized in the inward-facing state by these mutations, and used this information to guide an AI-based protein structure prediction tool to generate the inward open conformation (Extended Data Fig. 7a)^39,40^. Based on this inward open conformation prediction, we suggest that the strictly conserved Gln50(M1) is the probable candidate to control the p*K*_a_ of Asp152 (Extended Data Fig. 7c). Despite being 8.2 Å away from aspartate in the structure, Gln50 shifted by movements of M1 to make hydrogen bonds to the Asp152 in the predicted inward open model (Fig. 2b and Extended Data Fig. 7c). Substituting Gln50 to alanine led to loss of transport activity, comparable in magnitude to D152N in oocytes (Fig. 2c). Mutating other residues in the vicinity either did not display any reduced transport activity (Cys74) or showed a somewhat reduced transport activity (Ser78 or Gln456), except for the substitution of Trp47, which led to loss of transport comparable to the Q50A mutant (Fig. 2c). In addition, similar to the D152N mutant, the Q50A mutant also showed the same loss of current response and pH dependence compared with wild-type SUC1 in liposomes (Fig. 2d). Based on this, we propose that Asp152 is the proton acceptor/donor responsible for proton translocation during symport that may involve Gln50 for controlling p*K*_a_ changes in the proton site.

### Sucrose binding is coupled to protonation

**Fig. 3:**
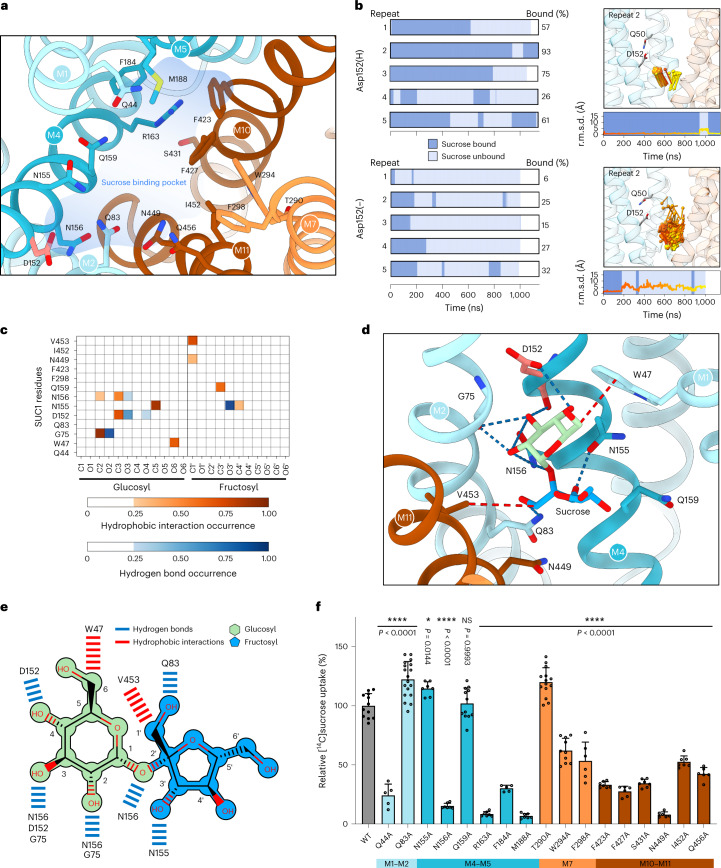
Identification and characterization of sucrose binding pocket. **a**, Close-up view of the sucrose binding pocket outlined in blue, as shown in Fig. 2a. **b**, Results from five independent MD simulation repeats each for Asp152(H) and Asp152(−), starting from the identified stable binding pose for sucrose. The repeats show consistent results, as summarized by the fraction of time in which sucrose was bound in the stable pose. Right, repeat 2 of the simulations is shown with sucrose as a pin, with the head representing the glucosyl moiety. Below, the r.m.s.d. plot of sucrose. Background of r.m.s.d. plot denoted bound (dark blue) or unbound (light blue) pose of the sucrose. **c**, Contact map between sucrose atoms and SUC1 residues in MD run 2 with Asp152(H). Only interactions occurring during at least 25% of the simulation time are shown, including hydrogen-bond interactions (blue) and hydrophobic interactions (red). **d**, Representative snapshot of the sucrose stable pose in simulations with Asp152(H). Blue dashes indicate hydrogen bonds and red dashes indicate hydrophobic interactions. **e**, Schematic representation of sucrose coordination by SUC1 in the snapshot shown in panel **d**. **f**, Oocyte uptake assay for SUC1 mutants targeting the residues lining the sucrose binding pocket. Uptake activities are normalized to that of the wild type. Bars are mean ± s.d. Points represent biologically independent experiments (*n* = 5 for Q44A and F184A; *n* = 6 for F298A, F427A, N449A and Q456A; *n* = 7 for N155A, N156A, R163A, M188A, F423A, S431A and I452A; *n* = 10 for W294A; *n* = 12 for WT and Q159A; *n* = 14 for T290A; *n* = 18 for Q83A). *P* values for differences between wild type and variants were obtained from a one-way analysis of variance (ANOVA) followed by the Dunnett’s multiple comparisons test. *P* values are shown in the figure. NS, not significant. Source data

From here we focused our interest towards the sucrose binding site located in the V-shaped cavity. There are clearly a range of residues from both the N and C domain in the cavity and close to the proton site that could participate in sucrose binding (Fig. 3a).

**Extended Data Fig. 10:**
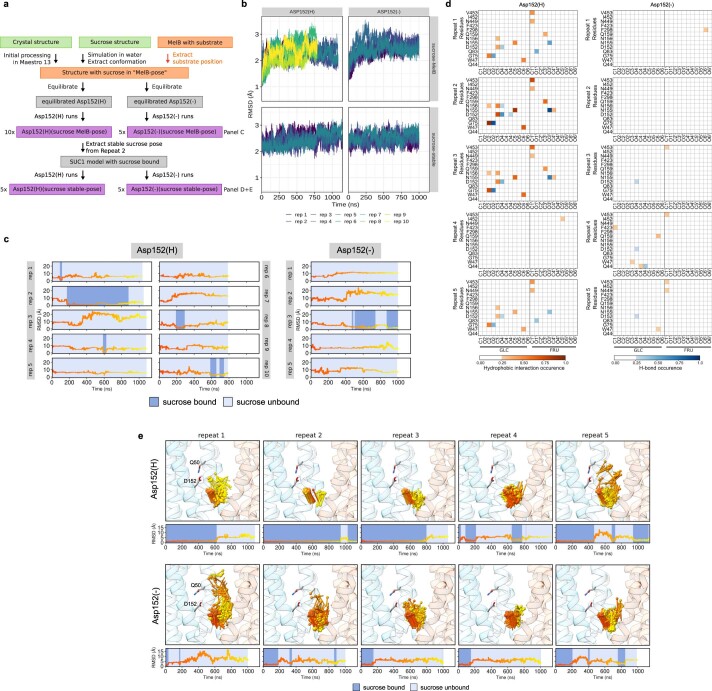
MD simulations. **a**, Flowchart of MD analysis. **b**, r.m.s.d. Cα backbone trace over the course of all MD simulations. All runs compared to the crystal structure. **c**, Comparison of time course of r.m.s.d. of sucrose from initial simulations performed with sucrose in the "MelB-inspired" starting pose, with Asp152 either protonated (Asp152(H)) or deprotonated (Asp152(−)). Stable binding of sucrose to SUC1 is denoted by dark blue background. **d**, Contact maps between sucrose atoms and SUC1 residues in all five repeats run with sucrose starting in the identified stable pose. Calculated contacts between atoms are defined by the interaction criteria provided by the ProLIF software^91^. Only interactions occurring in at least 25% of the frames are considered and includes hydrogen bond interactions (blue) and hydrophobic interactions (red). **e**, Comparison of time course of r.m.s.d. of sucrose from simulations performed with sucrose in the identified stable starting pose, also shown as a needle/pin with head representing the glucosyl moiety, with two different protonation states of Asp152: Asp152(H) and Asp152(−) in five independent repeats, respectively. Background of the r.m.s.d. plot denoted bound (dark blue) or unbound (light blue) position of the sucrose.

To assess the binding mode of sucrose and the functional roles of the residues in the central binding pocket, MD simulations with a sucrose molecule were performed to identify a stable binding pose of sucrose. For guidance, a starting pose for sucrose was based on the closest structural homologue known: the melibiose/cation symporter MelB bound with a galactosyl-containing melibiose analogue, which is proposed to mimic a substrate-bound state (15.7% sequence identity) (Extended Data Fig. 10a). We performed ten and five independent simulations of ~1 µs each (14.3 µs in total) on SUC1 with either a protonated Asp152 (Asp152(H)) or a deprotonated Asp152 (Asp152(−)) and a sucrose placed in the MelB-inspired pose. In all repeats, the protein showed a stable behaviour (Extended Data Fig. 10a,b).

Most of these simulations did not capture a static sucrose binding pose and sucrose would move freely around the cavity. However, in one of the repeats with Asp152(H), the glucosyl moiety of the sucrose stabilized in a static pose towards the N domain for over 1,050 ns (Extended Data Fig. 10c). To examine whether this pose resembles a stable initial binding event of the sucrose molecule, and if this is dependent on Asp152 being protonated, five new simulations with Asp152(H) or Asp152(−) (10.6 µs in total) were performed with sucrose placed in this new binding position (Extended Data Fig. 10d). In four of five repeats with Asp152(H), the sucrose maintained a stable binding pose, whereas it was readily and rapidly released in the Asp152(−) repeats (Fig. 3b and Extended Data Fig. 10e). Also, in the repeats with Asp152(H), the sucrose would return to the identified binding pose after a dissociation event. Mapping the contacts from the independent simulations shows that, in this initial stable binding pose, sucrose interacts specifically and directly with its glucosyl moiety at the Asp152(H) and residues Trp47, Gly75, Asn155 and Asn156 (Fig. 3c–e and Extended Data Fig. 10d). The contact analysis shows clearly that there are no other binding sites that are remotely comparable in scale. The contact map shows that hydrogen bonds are formed with the glucosyl hydroxyls at positions O2, O3 and O4, and this confers substrate recognition. In addition, hydrophobic interactions contribute to binding with the glucosyl and, to some extent, also the fructosyl initially. Altogether, the simulations pinpoint the glucosyl group of sucrose as the trigger of the initial binding event, creating initial selectivity, and hence suggest that a Asp152(H) is crucial for stabilizing sucrose binding directly within the transporter.

### Sucrose binding requires initial glucosyl moiety recognition

To test the interactions suggested by MD and probe their functional importance for transport, we next determined the biochemical characterizations of the proposed residues and other residues lining the cavity (Fig. 3a,d–f). Our results confirm that the residues predicted to be central for initial binding are essential for transport. Mutating Asn156 led to complete loss of transport in oocytes, consistent with its prominent role in coordinating the glucose part of sucrose (Fig. 3f). For the glucosyl moiety, the contact map also identified the backbone of Gly75, as well as the side chains of Trp47 and Asp152(H), leading to loss of transport activity when mutated (Fig. 2c). For the fructosyl contacts identified, mutagenesis of Asn155 had little apparent effect on transport activity, indicating the hydrogen bond to this residue, and any specific interactions to the fructosyl moiety in the initial binding pose, is of less importance for overall transport. For the other contacts identified, mutation of Gln159 had no effect, whereas mutating Asn449 eliminates transport (Fig. 3c,f). We also targeted residues in the identified binding site that was close to, but not yet engaged in, sucrose binding (Fig. 3a). Mutation of the polar residues Gln83 and Thr290 had no negative effects, whereas mutating the polar residues Gln44, Ser431 and Gln456 substantially reduced transport (Fig. 3f). In addition, mutagenesis of the hydrophobic residues Phe184, Trp294, Phe298, Phe423, Phe427 or Ile452 likewise reduced transport (Fig. 3f). Of interest, mutation of Arg163 or Met188 led to complete loss of transport, similar to Asn449 which is also located at the bottom of the cavity, together indicating a role for these three residues in the fully occluded state (Fig. 3f). Together, these data support that the stable sucrose binding pose observed in the MD simulations of protonated SUC1 represent an initial binding event. Initial substrate recognition by SUC1 is focused on hydrogen bonds to the glucosyl moiety, whereas other residues constituting the central binding pocket have roles in later stages of the transport cycle.

**Fig. 4:**
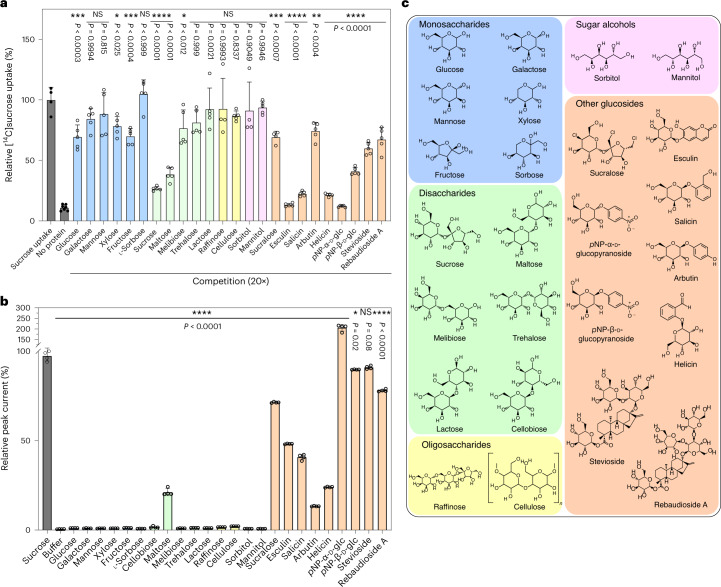
Substrate specificity of SUC1. **a**, Substrate specificity of SUC1 as determined by competition assay in oocytes with 1 mM sucrose and 20-fold of the competing substrates at pH 5.5. *P* values for differences between control (sucrose uptake without competition) and sucrose uptake with various competing compounds were obtained from a one-way ANOVA followed by Dunnett’s multiple comparisons test. *P* values are shown in the figure. Bars are mean ± s.d. Data points are individual experiments (*n* = 4 for sucrose uptake, sucralose, raffinose, sorbitol; *n* = 5 for all other compounds; *n* = 7 for no protein). **b**, Substrate specificity of SUC1 measured at pH 5.5 by SSM-based electrophysiology. Peak currents elicited by a range of putative substrates tested at 10 mM. Peak currents were normalized relative to sucrose-elicited currents after artefact signal correction. *P* values for current responses were obtained from a one-way ANOVA followed by Dunnett’s multiple comparisons test. *P* values are shown in the figure. Current responses indicated with * differed significantly from sucrose indicating that they are probably not ligands for the transporter, elicit lower peak currents or elicit higher peak current responses than sucrose. Bars are mean ± s.d.; *n* = 4. The points represent individual measurements and all measurements were performed on a single sensor. **c**, Chemical structures of glycosides used for screening of substrates. The screening includes monosaccharides (blue), disaccharides (green), oligosaccharides (yellow), sugar alcohols (pink) and other glucosides (orange). *p*NP-α-d-glc, *p*-nitrophenyl alpha-d-glucopyranoside; *p*NP-β-d-glc, *p*-nitrophenyl beta-d-glucopyranoside. Source data

To further support these results, we tested the substrate specificity of SUC1 with a wide range of different putative ligands, using both oocyte assays and SSM-based electrophysiology (Fig. 4a–c). We tested molecules both with and without a glucosyl and found that a glucosyl moiety is a required asset for substrates, whereas the fructosyl moiety can be substituted more readily with cyclic groups that have partial polar properties (Fig. 4). These results are consistent with the binding model that SUC1 interacts with the glucose unit of the sucrose in the initial stable binding pose, and that specific recognition of the fructose unit is less important for transport activity.

## Discussion

Our structural analysis, functional studies and MD simulation data provide a model for how sucrose is recognized by SUC1, and identify key structural elements, such as the proton-binding site, that are required for function. The presumed proton acceptor/donor Asp152 is strictly conserved in members of the SUT/SUC family (Extended Data Figs. 1 and 6) and has been reported previously as critical for sucrose-induced transport currents of SUC1 and for the corresponding rice ortholog OsSUT1 (refs. ^31,41^). SUC1 displays clear differences from other known proton-coupled symporters and presents a unique model for symport. First, the direct coupling between proton site and substrate binding site. Second, Gln50 on the M1 helix that we suggest controls the p*K*_a_ of Asp152 on the M4 helix, breaking with the generic pattern of using a basic residue for this function as observed in other proton transporters^14,35,42^. Because of these two factors, sugar binding becomes linked directly to proton binding, allowing sugar release into an environment with very high sucrose concentration, as long as the proton dissociates. The set up suggests a tight 1:1 coupling ratio between proton and sucrose, in accordance with previously reported kinetic models^34,43^. Our data suggest that the protonation of Asp152 is necessary to allow a tight polar interaction from the N domain to the glucosyl moiety of the substrate.

This tight and direct coupling provides a central pivot for explaining how members of the SUT/SUC family can operate at extreme molar-level sucrose concentrations. The low affinity of SUC1 is a prerequisite for high-capacity sucrose transport and is evident from the challenges in capturing a substrate-bound state in both crystallography and MD. Nevertheless, our work suggests that initial recognition of sucrose is mediated by interactions of the glucosyl to the protonated N domain. This is in agreement with previous findings that the glucosyl hydroxyl groups are central to substrate recognition^44–47^. We also find that residues in both N and C domains have important roles in the central binding pocket during the full transport cycle. The distribution of the contributing residues divides the binding pocket into two halves: one half contributes with polar interactions, mainly from the N domain; whereas the second half displays hydrophobic properties, mainly from the C domain (Fig. 3a). This is in contrast to the binding mode of glucose transporters such as animal glucose transporters (GLUTs) and plant Sugar Transport Proteins (STPs) from the Sugar Porter family. Here the C domain mediates most contacts through polar interactions to the monosaccharide^36,42,48,49^. In the proton-driven STPs, the N domain indirectly links a distal proton site to the high-affinity binding of glucose at the central binding site^36^.

In the SUC1 structure, the perfectly conserved Arg163 at the bottom of the binding cavity has a stabilizing gate-like role by forming hydrogen bonds to the backbone of Phe423(M10) and the side chain of Asn181(M5). Substitution to a lysine failed to complement transport activity, indicating a central role of the arginine in transport (Extended Data Figs. 6 and 7). In addition, the amphipathic helices of the EHR, stabilized by a critical disulfide bridge, and the amphipathic helix in the IHR are distinct structural features. Disrupting the IHR in oocyte assays had no apparent effect, whereas disrupting the EHR eliminated activity. Previous studies found that oxidizing agents increase transporter activity, whereas *p*-chloromercuribenzenesulfonic acid, which covalently binds to the SH group of cysteines, strongly inhibits transport activity^5^. These observations have been proposed to be related to dimerization of SUCs. Our results do not directly address possible dimerization, and the previous observations about transport activity can be explained fully by the key role we observe of the disulfide bridge at the EHR. However, the exact role of these amphipathic regions and any relation to possible dimerization remains unclear (Extended Data Fig. 5). The SUC1 architecture also suggests a physiological function of the linear dependence on pH and membrane potential that SUC1 transport activity exhibits^6,34,50^. This dependence is different when compared to SUC2, for example, which has a canonical pH optimum around pH 5.5 (refs. ^6,51^). As SUC1 is linked to sink tissues, where rapid postphloem sucrose transport is required to control sink strength, a direct 1:1 dependence on proton binding for substrate transport is meaningful. The tight proton coupling observed in SUC1 means that the driving substrate concentration, the proton concentration, is linearly linked to sucrose translocation, and allows release against a high concentration gradient^52^. Changing the apoplast pH may be used in a responsive manner by the plant to regulate and optimize the transporter activity of SUC1, which is supported by the low passive buffer capacity of the apoplast^51^. Acidifying the apoplast when competing with sugar transporters of invasive pathogens would increase both SUC1 transport capacity and apparent affinity, while simultaneously facilitating reduction of pathogen transporter activity.

The GPH family is a monovalent cation-driven symporter family and our work explains how this architecture has been be modified in plants to accommodate proton-driven transport. In the known structures of the bacterial melibiose/Na^+^ symporter MelB and the animal lysophosphatidylcholine/Na^+^ symporter MFSD2A^21,53,54^, residues at the equivalent position to Asp152 form part of the conserved sodium binding site, and MelB even displays cation promiscuity that, in part, determines substrate selectivity. The human solute carrier family 45 (SLC45) are also related to the SUT/SUC family, and relevant for melanin synthesis and a range of correlated diseases. In SLC45A2, mutation of the residue equivalent to Asp152 causes oculocutaneous albinism, again supporting the critical role of the aspartate in orthologs across kingdoms^55^. Furthermore, the equivalent residue to Arg163 plays an important role in substrate binding in the ligand-bound MelB structure, and mutating it in mammalian MFSD2A is lethal, once more demonstrating the key role of this position within the GPH family^54,56^.

**Fig. 5:**
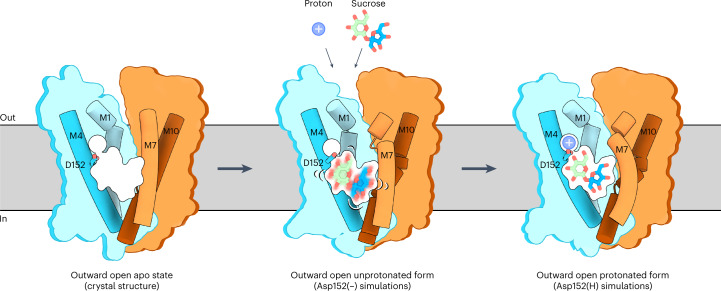
Proposed mechanism of substrate recognition in SUC1 transport. The N and C domains are coloured according to the SUC1 structure, with highlighted M1, M4, M7 and M10 shown as cartoons. Sucrose is shown in green and blue and the proton is shown as a blue sphere.

Based on our data we propose that SUCs operate by an alternating-access mechanism with the following mechanistic model for substrate recognition in transport (Fig. 5). In the outward open state, a large cavity is open towards the apoplastic side that allows sucrose and proton entrance. The sucrose settles with the glucosyl moiety nested against Asp152 and surrounding residues (Fig. 3d–e). This initial low-affinity binding to the glucosyl moiety is dependent on the protonation of Asp152. After sucrose and proton binding, Arg163 and other binding pocket residues create further sucrose contacts in later stages of substrate recognition. This forms a two-step recognition scheme involving an initial glucosyl recognition site and later involvement of Arg163 in sucrose translocation, consistent with previous studies^57^. This recognition setup also explains the broader substrate promiscuity toward the secondary saccharide of the substrate. Although the glucosyl is relatively invariant, the fructosyl can be exchanged more readily (Fig. 4). During transporter transition, the intracellular network of salt bridges is broken, whereas, at the extracellular side, charged and polar residues probably form contacts between helices to ensure closure of the cavity from the apoplastic side and stabilization towards the inward state, as supported by predicted models and oocyte mutagenesis studies (Extended Data Fig. 7a,b). After opening to the inside, p*K*_a_ changes directed by Gln50 lead to deprotonation of Asp152 to favour sucrose release.

In conclusion, our work pinpoints Asp152 as the proton-binding site in the SUT/SUC family and shows how sucrose binding, with initial recognition of the glucosyl, is achieved through a direct coupling to proton binding at this site. We identify key elements and properties of transporter function and explain a central molecular mechanism behind sugar signalling, stress responses and the central driving force behind vascular movement of sucrose and other solutes in vascular plants.

## Methods

### Protein purification

*A. thaliana* SUC1 (UniProt: Q39232) cDNA was introduced into an expression construct based on p423-GAL1 with a C-terminal thrombin cleavage site and a decahistidine purification tag. Transformed *S. cerevisiae* (strain DSY-5) were grown in a culture vessel to high density by fed-batch cultivation and harvested after a 22 h induction using galactose^58^. Harvested cells were washed in cold water, spun down and resuspended in buffer (100 mM Tris pH 7.5, 600 mM NaCl, 1.2 mM phenylmethylsulfonyl fluoride) followed by bead beating with 0.5 mm glass beads for lysing. The homogenate was centrifuged for 20 min at 17,568*g*, followed by sedimentation of membranes by ultracentrifugation at 200,000*g* for 2 h. Cell membranes were homogenized and resuspended in buffer (50 mM Tris pH 7.5, 500 mM NaCl, 20% glycerol) before being frozen in liquid nitrogen and stored at −80 °C. Thawed cell membrane was solubilized in buffer (150 mM NaCl, 50 mM Tris pH 7.5, 5% (v/v) sucrose) with 1% (w/v) *n*-dodecyl-*β*-d-maltoside (DDM) and 0.1% (w/v) cholesterol hemisuccinate (CHS) for 30 min at 4 °C. Insoluble material was removed by centrifugation at 5,000*g* for 10 min, and the supernatant was filtered using a 1.2 μm filter (PALL). The supernatant was supplemented with 20 mM imidazole (pH 7.5) and loaded on a 5 ml Ni-NTA column (GE Healthcare) at 3 ml min^−1^. The column was washed with ten column volumes of buffer (solubilization buffer supplemented with 0.1% DDM, 0.01% CHS and 90 mM imidazole pH 7.5) and 20 column volumes of buffer (20 mM Tris pH 7.5, 250 mM NaCl, 5% (v/v) sucrose, 0.02% (w/v) DDM, 0.002% (w/v) CHS and 0.5 mM tris(2-carboxyethyl) phosphine (TCEP)) supplemented with 40 mM imidazole (pH 7.5). Protein was eluted in the same buffer containing 500 mM imidazole (pH 7.5) and eluted protein samples were pooled, supplemented with bovine thrombin followed by dialysis overnight at 4 °C in buffer (20 mM 2-(*N*-morpholino)ethanesulfonic acid (MES) pH 6.0, 250 mM NaCl, 5% (v/v) sucrose, 0.02% (w/v) DDM, 0.002% (w/v) CHS and 0.5 mM TCEP) using 12–14 kDa molecular weight cut-off (MWCO) tubing (Spectrum). On the next day, the protein was supplemented with 50 mM Tris (pH 7.5) and loaded on a 5 ml Ni-NTA column (GE Healthcare) to remove the purification tag and contaminants. Protein was collected with buffer (20 mM Tris pH 7.5, 250 mM NaCl, 5% (v/v) sucrose, 0.02% (w/v) DDM, 0.002% (w/v) CHS and 0.5 mM TCEP) supplemented with 40 mM imidazole (pH 7.5), concentrated using a 50 kDa MWCO spin column (Vivaspin) and subjected to Superdex 200 Increase 10/300 GL column (GE Healthcare) in buffer (20 mM MES pH 6.0, 250 mM NaCl, 5% (v/v) sucrose, 0.02% (w/v) DDM, 0.002% (w/v) CHS and 0.5 mM TCEP). The composition of the size-exclusion buffer was optimized for sample stability^59^. Peak fractions were pooled and the protein was concentrated to 14 mg ml^−1^ using a 50 kDa MWCO spin column (Vivaspin).

### Crystallization

SUC1 was crystallized in the lipidic cubic phase. Purified protein was mixed with monoolein (Sigma) in a 1:1.5 protein-to-lipid ratio using a syringe mixer. For crystallization, 50 nl of the meso phase were mixed with 1000 nl of crystallization buffer containing 43.5% polyethylene glycol 400 (v/v), 0.25 M ammonium dihydrogen phosphate and 0.15 M bis-tris propane (pH 6.3) on glass sandwich plates using a Gryphon robot (Art Robbins Instruments). The lipidic cubic phase crystals appeared after 1–2 days and grew to full size of 100 × 10 × 30 µm at 19 °C within 1 week. The crystals were collected using Dual Thickness MicroMounts LD (MiTeGen) and flash frozen in liquid nitrogen.

### Data collection and structure determination

After extensive screening, the final dataset was collected at Diamond Light Source microfocus beamline I24 using a 20 × 20 µm beam. A single crystal was diffracted for 360° with a 0.10° oscillation angle with 1.0 Å wavelength X-rays. The dataset was processed and scaled using DIALS^60^ and AIMLESS^61^ through the XIA2 pipeline^62^ in triclinic space group *P*1 (point group 1), which suggested the presence of two SUC1 molecules in the asymmetrical unit with ~56% solvent content. In attempts to solve the phase problem, 65 search models generated from known structures of MFS members were used for molecular replacement. However, no solution suitable for structure solution could be obtained. The phase problem was solved by molecular replacement using PHASER^63^ with a RoseTTAFold-predicted^64^ SUC1 model; r.m.s.d. estimates of the model were converted into B-factors before final modifications of the search model using SCULPTOR^65^. This search gave a solution with a translation-function *Z* score of 9 and log-likelihood gain score of 259 at a 3.5 Å resolution cutoff, with two molecules in the asymmetrical unit forming a non-physiological dimer with an initial *R*_free_ calculation of 52% in Refmac^66^. Initial refinement with Refmac and density modification with histogram matching, solvent flattening and anisotropy correction was done to reduce search model bias with Parrot^67^ in the CCP4 suite^68^. The electron density map allowed for iterative model building in Coot^69^ and refinement using phenix.refine using 2mFO-DFc maps and generated Feature Enhanced Maps^70^ using model phases. We performed final refinement in phenix.refine with a refinement strategy of individual sites, individual atomic displacement parameters (ADP) and group translation–libration–screw (nine groups) and non-crystallographic symmetry restraints against a maximum likelihood target with reflections in the 35–2.68 Å resolution range. The final model yielded a free *R* factor (*R*_free_) of 29.30% and an *R* factor (*R*_work_) of 26.96% (Supplementary Table 1). MolProbity^71^ evaluation of the Ramachandran plot gave 98.31% in favoured regions and 0.13% outliers. Figures were prepared using ChimeraX v.1.4 (ref. ^72^). Sequence alignments were constructed with PROMALS3D^73^. Alignments were visualized using ALINE v.1.0.025 (ref. ^74^). Evolutionary sequence conservation was analysed using ConSurf^75^. The coulombic electrostatic potential was calculated in ChimeraX v.1.4 (ref. ^72^).

### Preparation of liposomes and proteoliposomes

Soy polar lipid extract (composition (w/w), 45.7% phosphatidylcholine, 22.1% phosphatidylethanolamine, 18.4% phosphatidylinositol, 6.9% phosphatidic acid and 6.9% other soy lipids) in chloroform (Avanti) was dried and resuspended in reconstitution buffer (30 mM HEPES pH 7.4, 140 mM NaCl and 5 mM MgCl_2_). Liposomes were homogenized by extruding through 0.4 µm pore size polycarbonate membranes (Millipore) and *n*-octyl-β-d-glucoside (Anatrace) was added to a final concentration of 1% (v/v). Liposome suspension was sonicated in water bath three times for 1 min, with incubation on ice for 1 min in between each sonication. Purified protein was added to liposomes to a calculated lipid:protein ratio of five. The detergent *n*-octyl-β-d-glucoside was removed by incubating with 400 mg ml^−1^ Bio Beads (Bio-Rad) overnight at 4 °C using a rotary shaker. Proteoliposomes (5 mg ml^−1^) were flash frozen in liquid nitrogen and stored at −80 °C.

### SSM-based electrophysiology assays

SSM-based electrophysiology was performed on a SURFE2R N1 (Nanion Technologies). For all experiments, preparation of sensors was performed as described^76^. Proteoliposomes were diluted 1:5 in non-activating buffer, sonicated three times for 10 s in a water bath and applied to 3 mm sensors followed by centrifugation for at 2,100*g* for 30 min at 4 °C. For sugar titration assays, both non-activating and activating buffer were prepared from the same main buffer (5 mM MgCl_2_ and 100 mM potassium phosphate buffer at a given pH value). Non-activating buffer was supplemented with 810 mM mannitol, a structurally related molecule which is not transported by SUC1. Activating buffer was prepared in an equimolar manner by adding 810 mM sucrose, followed by dilution in non-activating buffer to keep the osmolarity constant for all solutions used during one experiment. A single solution exchange workflow was used. Data points represent the mean of three independent measurements. For substrate specificity measurements, non-activating and activating buffer were prepared from main buffer pH 5.5, with the activating buffer containing 10 mM of the substrate of interest. The assay was performed on both sample and negative control membranes to distinguish currents originating from SUC1 and artefact currents induced by the different components interacting with the SSM. To determine SUC1 transport currents for each individual substrate, the measured currents for a given substrate had the average value of the corresponding negative control subtracted from it. The data represent the mean of three independent measurements performed on a single sensor and were normalized according to sucrose measurements. For the measurement under symmetrical pH conditions, a single solution exchange configuration was used. The sample was incubated at a given pH for 6 min between measurements at different pH values to adjust the inner pH of the proteoliposomes to the external pH. Peak currents were measured upon addition of activating buffer at the given pH containing 10 mM sucrose. For each sensor, after measurements at different pH values, the signal of the sensor at the initial neutral pH was measured again to evaluate potential loss of signal during the pH titration, for instance, caused by diminishing protein stability at different pH. These signal amplitudes were comparable to initial measurements, demonstrating that transporter activity was retained throughout the experiment. Experiments were performed at least in triplicate and data were analysed with GraphPad Prism v.9. Michaelis–Menten fitting was used for curve fitting analysis and *K*_m_ determination. Error bars represent the s.d. As the peak current signal can be a combined effect of electrogenic events from binding, transport and shielding or neutralization of currents, we use the *K*_m_^app^ parameter only for sucrose transport throughout the manuscript, which may alternatively also be described as the half maximal effective concentration. In symport we have more than one substrate, in this case, sucrose and H^+^. For any two-substrate cases, the *K*_m_ value for one substrate is, in principle, determined by extrapolation to infinite concentrations of the second substrate. Thus, we use ‘app’ in *K*_m_^app^ to emphasize that the *K*_m_ values determined for sucrose apply specifically at the defined pH.

### Oocyte uptake assays

*A. thaliana* SUC1 (UniProt: Q39232) cDNA was cloned into the pNB1-U vector^77^ and GFP fusion variant of the vector harbouring a C-terminal ten residue glycine–serine linker followed by the gene sequence encoding eGFP. All mutations were created using site-directed mutagenesis with a Q5 polymerase (New England Biolabs). For cDNA preparation, genes were amplified by PCR using standard primers with 5′ and 3′ untranslated regions (forward, TTAACCCTCACTAAAGGGTTGTAATACGACTCACTATAGGG; reverse, TTTTTTTTTTTTTTTTTTTTTTTTTTTTTATACTCAAGCTAGCCTCGAG) followed by PCR purification (GeneJET PCR Purification, Thermo Fisher) after separation on 1% agarose gel. RNA was synthesized by in vitro transcription using the mMESSAGE mMACHINE T7 Transcription Kit (Thermo Fisher). Capillary needles for RNA injection were made using a PC-10 micropipette puller (Narishige). For expression, *Xenopus laevis* oocytes (EcoCyte Bioscence) were each injected with 25 ng of RNA using the Nanoject III (Drummond Scientific), followed by incubation at 16 °C for 3 days in ND-96 buffer (96 mM NaCl, 1 mM MgCl_2_, 2 mM KCl, 1.8 mM CaCl_2,_ 5 mM HEPES pH 7.4) with 10 IU ml^−1^ gentamycin (Invitrogen). For concentration-dependent uptake assays, concentrations of sucrose ranging from 0.1 to 20 mM in ND-96 reaction buffer (96 mM NaCl, 1 mM MgCl_2_, 2 mM KCl, 1.8 mM CaCl_2,_ 5 mM MES pH 5.5) was used and [^14^C]sucrose (PerkinElmer) was added to each sugar solution at a concentration of 1 μCi ml^−1^. Before uptake assays, oocytes were washed and pre-incubated for 5 min in reaction buffer. For the uptake assay, oocytes were incubated in the reaction buffer at a given sucrose concentration for 30 min. All assays were done at pH 5.5 unless specifically noted. The reaction was stopped by adding ice-cold reaction buffer supplemented with 100 mM sucrose followed immediately by washing of the oocytes in ice-cold reaction buffer. For controls, oocytes injected with pure water instead of RNA were incubated in a similar manner. In competition assays, the reaction buffer was composed of 1 μCi ml^−1^ [^14^C]sucrose, 0.5 mM sucrose and a 20-fold higher concentration of the competing sugar (10 mM). For uptake activity assay of mutants and pH-dependent uptake assays, the reaction buffer was composed of 1 μCi ml^−1^ [^14^C]sucrose and 1 mM sucrose. For the pH-dependent assays, the reaction buffer was supplemented with 50 mM potassium phosphate adjusted to the intended pH, or 25 mM citrate adjusted to pH 3.5, 4.5 or 5.5 in pH-dependency assays of mutants. For time-dependent uptake assays, a concentration of 675 μM sucrose (Sigma-Aldrich) was used and [^14^C]sucrose was added to a final concentration of 1 μCi ml^−1^. Each oocyte was treated as a single experiment. The oocytes were transferred individually to a scintillation vial and disrupted by the addition of 100 μl of a 10% SDS solution followed by immediate vortexing. A volume of 3 ml OptiPhase HiSafe 3 scintillation fluid (PerkinElmer) was added to each sample, and radioactivity was quantified using a Tri-Carb 4910TR Liquid Scintillation Counter (PerkinElmer). Experiments were performed at least in triplicate and data were analysed using GraphPad Prism v.9. Michaelis–Menten fitting was used for curve fitting analysis and *K*_m_ determination. Error bars represent the s.d. To determine if protein localization was affected by point mutagenesis, cellular localization was analysed on C-terminal eGFP-fused wild types and mutants by confocal laser scanning microscopy. Images were captured using a Zeiss LSM 780 Axio Imager 2 confocal laser scanning microscope with the operating software ZEN v.3.5. We used the 10× objective with an excitation wavelength of 488 nm using 2.55% laser power with a frame time of 7.75 s, displaying a quarter of an optical slice of a 3-day-postinjection oocyte. Because we have more than one substrate in symport, *K*_m_^app^ values were used as explained in SSM-based electrophysiology assays.

### Model prediction pipeline using the AI-based protein structure prediction tool AlphaFold v.2

The predicted model from RoseTTAFold, which we used for molecular replacement phasing, and the predicted model from AlphaFold v.2, both display an outward open conformation with r.m.s.d. of Cɑ atoms to the experimental structure between 1.6 and 2.5 Å. Thus, the AI-predicted models are quite accurate, at least for the core parts of the structure. To identify residues that could be responsible for affecting the p*K*_a_ of Asp152 to direct protonation state, we used AlphaFold v.2 (ref. ^39^) to sample alternative conformations of SUC1. This was done by reducing the depth of the input multiple sequence alignment (MSA)^40^ and modifying the SUC1 input protein sequence by introducing alanine mutations in Arg99, Arg100 and Arg357, which are residues of the intracellular network of the interdomain salt bridge network that stabilizes SUC1 in the outward open conformation. Prediction runs were executed using AlphaFold v.2.0.1 through the ColabFold server using the AlphaFold2_advanced notebook^78^. All MSAs were obtained using the MMSeqs2 server^79^ and the max_msa_clusters was reduced to 32 randomly chosen sequence clusters and max_extra_msa was reduced to 64 extra sequences that were used to compute additional summary statistics. All predicted models were subjected to Amber-Relax following their prediction.

### MD model preparation

Chain A of the crystal structure of SUC1 was initially processed using Maestro v.13.0. The protonation states of ionizable residues were determined using PROPKA^80^, where the apoplastic side of the transporter was at pH 5, and the cytoplasmic side was at pH 7. Hydrogen bonding was optimized when considering the choice of tautomers. Asp304 (p*K*_a_ 7.47) was modelled as protonated; His65 (p*K*_a_ 6.19), His205 (p*K*_a_ 5.90) and His390 (p*K*_a_ 6.25) were exposed to the apoplastic milieu and were, therefore, protonated on both Nδ and Nε of the imidazole ring. His89 (p*K*_a_ 6.14) faced the cytoplasm and was protonated only on Nε. Asp152 (p*K*_a_ 6.50), located in the central cavity, was considered both in a protonated (Asp152(H)) and a deprotonated (Asp152(−)) state. The observed disulfide bridge was modelled between Cys216 and Cys220.

SUC1 was aligned with the *z* axis and placed in a cubic box (90 × 90 × 118 Å^3^) and surrounded by 188 1-palmitoyl-2-oleoyl-glycero-3-phosphocholine lipids in an *x*–*y* plane. The protein–membrane system was solvated in transferable intermolecular potential with 3 points (TIP3P) water^81^ and a charge-neutralizing 150 mM NaCl.

Initial sucrose coordinates were accessed through the Research Collaboratory for Structural Bioinformatics Protein Data Bank (chemical identifier PRD_900003). To explore the conformational landscape, a single sucrose molecule was placed in a dodecahedron box, solvated with TIP3P water^81^ and 0.15 M NaCl and simulated using the CHARMM36m force field^82^ in three repeats and for 1 μs each.

The conformational poses of sucrose resulting from these simulations were clustered using the gromos algorithm^83^ implemented in Gromacs analysis suite^84^ with a clustering cutoff value of 4 Å. The middle structure of the biggest cluster, consisting of half of all simulation frames, was taken as a representative sucrose pose. Placement of this sucrose pose into the central cavity of SUC1 was informed by a bacterial homologue melibiose permease MelB (PDB: 7L17 that was cocrystallized with 4-nitrophenyl α-d-galactopyranoside^54^. First, MelB and SUC1 structures were aligned based on the positions of backbone Cα atoms. Next, ring carbons of the glucosyl moiety of sucrose were aligned with the ring carbons of galactose present in the MelB structure. SUC1 was modelled with two protonation states of Asp152: Asp152(−), resulting in a negatively charged side chain, and the Asp152(H) (uncharged) state. The simulations for both systems were initiated with this MelB-inspired identical sucrose pose (Extended Data Fig. 10a). After ~14 μs of simulations, the simulation that exhibited the most stable binding of the substrate (Extended Data Fig. 10c), repeat 2 of Asp152(H), was used to extract a stable sucrose binding pose in the central cavity of SUC1. The simulation trajectory was aligned to the initial Cα atoms of the protein backbone, the gromos clustering algorithm^83^ was used on the r.m.s.d. values of sucrose heavy atoms with a cutoff value of 3.8 Å and the middle structure of the biggest cluster (representative of 78% of frames of the trajectory) was used as a conformation of the stable sucrose binding pose in SUC1. A second round of simulations was now initiated from the identified stable sucrose pose and under the two protonation conditions of Asp152 (Extended Data Fig. 10a).

### MD simulation protocol

All simulations were performed with the CHARMM36m force field^82^ and in Gromacs 2020 simulation software^84^. Initially, the systems were minimized using the steepest descent algorithm until forces converged or reached a maximum value of 500 kJ mol^−1 ^nm^−1^. Temperature was set to 296 K to reflect the *A. thaliana* growing conditions. Temperature was regulated using the Berendsen thermostat^85^ during the equilibration stage, and a velocity-rescaling thermostat^86^ during the production run, with the temperature coupling applied on three groups: protein with the substrate, membrane and solvent. For the sucrose-only simulations, there were only two coupled groups: sucrose and solvent. The pressure of 1 atm was maintained using the Berendsen barostat^85^ during equilibrations, and the Parrinello–Rahman barostat^87^ during the production runs. All simulations containing a membrane had a semi-isotropic pressure coupling type, whereas sucrose-only simulations were simulated in an isotropic manner. During the production runs, the leapfrog algorithm integration step was set to 2 fs, whereas the LINCS constraint algorithm^88^ was applied to all bonds associated with hydrogen atoms in the system. Computation of long-range electrostatic interactions was performed using the particle mesh Ewald method^89,90^, with a 1.2 nm cutoff for real-space interactions. The cutoff value for Van der Waals interactions was set to 1.2 nm, with a force-switch modifier applied at 1.0 nm. All systems were equilibrated using the standard CHARMM-GUI protocol and with gradually decreasing position restraints, starting from 4,000 kJ mol^−1 ^nm^−2^ for the backbone atoms and sucrose ring atoms; 2,000 kJ mol^−1 ^nm^−2^ for the protein side chain atoms and remaining sucrose heavy atoms; and 1,000 kJ mol^−1 ^nm^−2^ for the phosphorus atom of each lipid, the select dihedral angles of the glycerol moiety and the double-bonded segment of the lipid tail. Equilibrations were run for 250 ps in a canonical ensemble (*NVT*), followed by 1.75 ns of isothermal–isobaric ensemble (*NPT*) equilibrations. The production runs for SUC1 in membrane were performed without any position restraints, in repeats of five or ten and up to a microsecond timescale (Extended Data Fig. 10c).

### MD simulation analysis

The r.m.s.d. for the protein Cα atoms was calculated in Gromacs^84^ against the reference SUC1 crystal structure. The r.m.s.d. value for the sucrose was compared to the stable sucrose pose as a reference. If a sucrose pose had an r.m.s.d. value below or equal to 3 Å, the substrate was considered bound, whereas higher r.m.s.d. values designated the pose to be unbound. The protein–sucrose interactions were assessed using ProLIF software^91^, in which hydrogen bonds are defined as interactions between a donor and an acceptor involving a hydrogen, within the 3.5 Å distance and forming an angle between 130 and 180°. Hydrophobic interactions are between non-polar atoms placed at a distance below or equal to 4.5 Å. The averaged contact maps for Asp152(H) and Asp152(−) systems were generated by considering frames of all simulations starting from a stable sucrose pose, calculating the frequency of each interaction across all frames and visualizing only the interactions that occur for at least 25% of the total simulation time.

### Reporting summary

Further information on research design is available in the Nature Portfolio Reporting Summary linked to this article.

## Supplementary information

Supplementary Information Supplementary Table 1. Data collection and refinement statistics.

Reporting Summary

## Data Availability

Atomic model and structure factors were deposited in the Protein Data Bank (PDB: 8BB6). The MelB coordinates used for assigning initial sucrose position for MD can be downloaded from the Protein Data Bank (PDB:7L17). The SUC1 protein sequence for *A. thaliana* used in this study is publicly available at UniProt (https://www.uniprot.org/) (UniProt: Q39232). Source data are provided with this paper.

## Source data

Source Data Fig. 1 Uptake assay, raw counts per minute. SSM electrophysiology, peak currents.
Source Data Fig. 2 Uptake assay, raw counts per minute. SSM electrophysiology, peak currents.
Source Data Fig. 3 Uptake assay, raw counts per minute.
Source Data Fig. 4 Uptake assay, raw counts per minute. SSM electrophysiology, peak currents.
Source Data Extended Data Fig. 3 Uptake assay, raw counts per minute. SSM electrophysiology, peak currents.
Source Data Extended Data Fig. 5 Uptake assay, raw counts per minute.
Source Data Extended Data Fig. 7 Uptake assay, raw counts per minute.
Source Data Extended Data Fig. 8 Uptake assay, raw counts per minute. SSM electrophysiology, peak currents.
