# Supplementary material for: Structure and sucrose binding mechanism of the plant SUC1 sucrose transporter
Source: Nat Plants. 2023 May 15;9(6):938–50. doi: 10.1038/s41477-023-01421-0 (PMC10281868; doi:10.1038/s41477-023-01421-0)
Supplement: Supplementary file 1 — Supplementary Table 1. Data collection and refinement statistics. [file 41477_2023_1421_MOESM1_ESM.pdf]

nature plants

Article

https://doi.org/10.1038/s41477-023-01421-0

Structure and sucrose binding mechanism of the plant SUC1 sucrose transporter

In the format provided by the authors and unedited

Supplementary Table 1: Data collection and refinement statistics.

**Name**	**SUC1**
**State**	Outward open
**Data Collection**	
Space group	P 1
Cell dimensions	
a. b, c (Å)	58.78 65.62 82.81
alpha, beta, gamma (deg)	89.96 101.74 94.72
Monomers per asym. unit.	2
Wavelenght (Å)	1.0
Number of reflections measured	110,948
Number of unique reflections	33,404
Resolution (Å)	57.35-2.68 (2.73-2.68)[a]
Rmeas (%)	26.2 (166.6)
Mean I/σ(I)	4.7 (0.8)
CC(1/2)	97.6 (31.0)
Completeness (%)	98.6 (95.1)
Redundancy	3.3 (3.4)
**Refinement**	
Resolution (Å)	34.73 - 2.68 (2.78 - 2.68)
No. reflections (work/free)	33,275 / 3,242
Rwork (%)	26.96
Rfree (%)	29.30
No. of Atoms	
Protein	7188
Waters	34
Average B Factors (Å)	
Overall	48.4
Protein	48.4
Waters	43.8
RMSD	
Bond lenghts (Å)	0.0047
Bond angles (deg)	0.89
Validation	
MolProbity score	1.31
Clashscore	5.64
Poor rotamers (%)	0.13
Ramachandran Plot Statistics	
Favored regions	98.31
Allowed regions	1.69
Disallowed regions	0.00
Deposited model (PDB ID)	8BB6

[a] Highest resolution shell is shown in parenthesis.
